# Supplementary material for: mRNA Inventory of Extracellular Vesicles from Ustilago maydis
Source: J Fungi (Basel). 2021 Jul 14;7(7):562. doi: 10.3390/jof7070562 (PMC8306574; doi:10.3390/jof7070562)
Supplement: Supplementary file 1 [file jof-07-00562-s001.zip › Supplementary files revised/Figure S1.pdf]

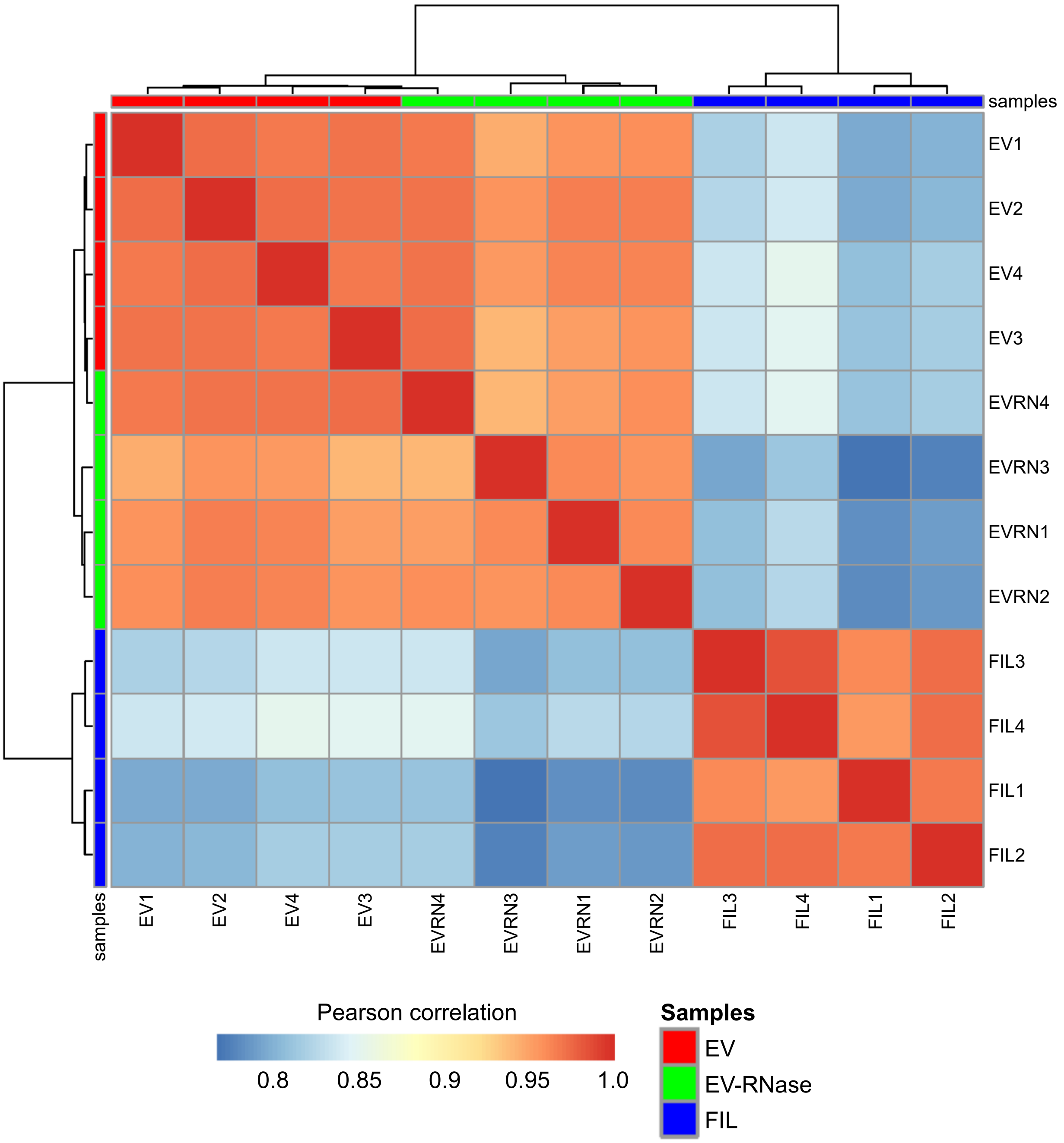

Figure S1. Heatmap showing Pearson correlation between the biological replicates of mock- and RNase- treated EV samples and the corresponding filament samples sequenced.
