# Supplementary material for: mRNA Inventory of Extracellular Vesicles from Ustilago maydis
Source: J Fungi (Basel). 2021 Jul 14;7(7):562. doi: 10.3390/jof7070562 (PMC8306574; doi:10.3390/jof7070562)
Supplement: Supplementary file 1 [file jof-07-00562-s001.zip › Supplementary files revised/Figure S2.pdf]

(a)

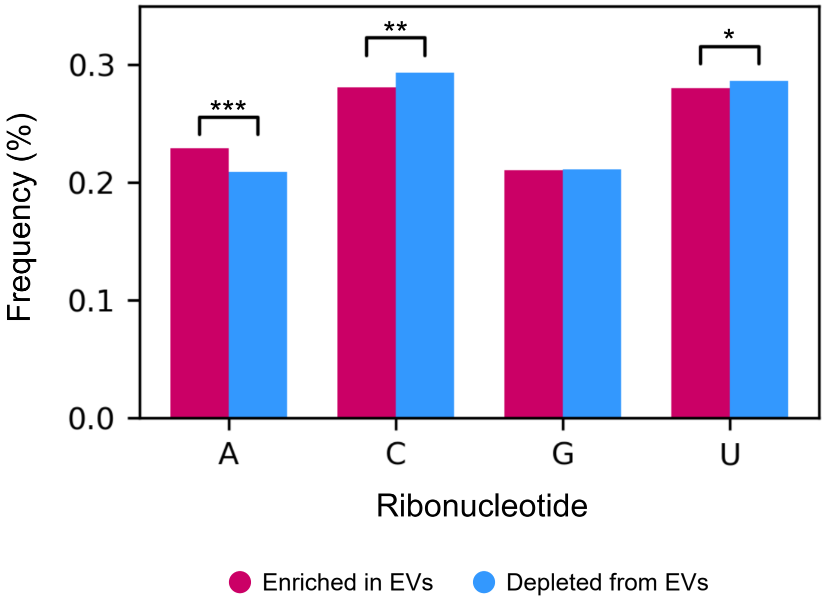

(b)

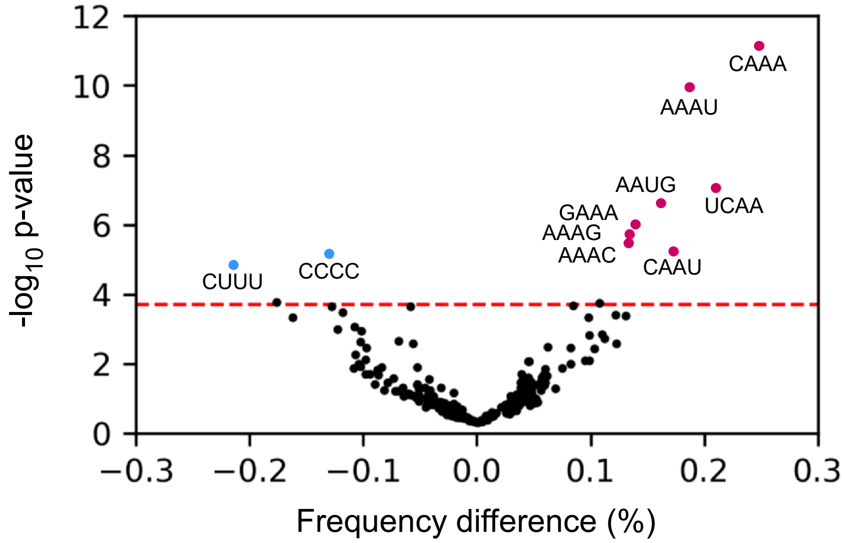

Figure S2. Overrepresented nucleotides and k-mers in the 3′UTRs of mRNAs enriched in EVs. (**a**) Single nucleotide frequency differences between enriched (red) and depleted (blue) sequences. Asterisks indicate significance (* p = 0.0096, ** p = 1.1 x 10-7, *** p = 1.3 x 10-19). (**b**) p-value (-log10) as a function of 4-mer frequency differences between enriched and depleted sequences. Positive values indicate increased frequency of a 4-mer in enriched UTRs than in depleted UTRs. 4-mers whose frequency differs significantly between enriched and depleted 3′UTRs are above the red dotted line ($p < 0.05$ and Bonferroni correction).
